# A New General Medical Journal with High Aspirations

**DOI:** 10.31662/jmaj.s0003

**Published:** 2018-09-28

**Authors:** Tsuguya Fukui, Yutaka Atomi

**Affiliations:** 1 St. Luke’s International University; 2 Kyorin University

It is our utmost pleasure to announce that we have now successfully launched the *JMA Journal*, a new official medical journal in English under the auspices of the Japan Medical Association (JMA) and the Japanese Association of Medical Sciences (JAMS). The JMA has been publishing its official English journal for 60 years to mostly serve as a source of information on medical situations in Japan. With a completely different look and content, and an open access journal, the *JMA Journal* is a high standard general medical journal carrying original and review articles covering a wide range of clinical practice, basic sciences and news of medicine, medical care, and health policy, among others.

It is truly fortunate to have a slew of leaders and senior researchers in a variety of medical fields from inside and outside of Japan to form the entirety of editorial board of the *JMA Journal*. As a result, we have a capacity to swiftly process with numerous articles submitted from all over the world and publish high quality articles without delay.

We have high aspirations to be regarded in the not-too-distant future as the leading medical journal from Japan for general readers all over the globe. It is envisioned that the *JMA Journal* serves as a pivotal source of trustworthy and up-to-date information from medical communities in Japan and other countries as JAMA from the United States of America and BMJ from the United Kingdom.

We sincerely solicit our readers to submit pertinent articles, making the *JMA Journal* the platform for discussions on medical issues of international relevance and we will see in the years to come many scientific achievements in medicine become more widely known internationally through the *JMA Journal*.

Tsuguya Fukui, MD, MPH, PhD

Yutaka Atomi, MD, PhD

Editors in Chief, JMA Journal

